# A Mobile App to Screen for Neurocognitive Impairment: Preliminary Validation of NeuroScreen Among HIV-Infected South African Adults

**DOI:** 10.2196/mhealth.9148

**Published:** 2018-01-05

**Authors:** Reuben N Robbins, Hetta Gouse, Henry G Brown, Andries Ehlers, Travis M Scott, Cheng-Shiun Leu, Robert H Remien, Claude A Mellins, John A Joska

**Affiliations:** ^1^ HIV Center for Clinical and Behavioral Studies Department of Psychiatry New York State Psychiatric Institute & Columbia University New York, NY United States; ^2^ HIV Mental Health Research Unit Department of Psychiatry and Mental Health University of Cape Town Cape Town South Africa; ^3^ Envisage IT Cape Town South Africa; ^4^ Department of Psychology Fordham University Bronx, NY United States

**Keywords:** HIV, neurocognitive, impairment, lay health workers, resource-limited settings, South Africa, tablet, app, neuropsychology

## Abstract

**Background:**

Neurocognitive impairment (NCI) is one of the most common complications of HIV infection, and has serious medical and functional consequences. South Africa has 7 million people living with HIV (PLHIV) with up to three-quarters of antiretroviral therapy (ART)-naïve individuals having NCI. South Africa’s health system struggles to meet the care needs of its millions of PLHIV; screening for NCI is typically neglected due to limited clinical staff trained to administer, score, and interpret neuropsychological tests, as well as long test batteries and limited screening tools for South African populations. Without accurate, clinically useful, and relatively brief NCI screening tests that can be administered by all levels of clinical staff, critical opportunities to provide psychoeducation, behavioral planning, additional ART adherence support, and adjuvant therapies for NCI (when they become available) are missed. To address these challenges and gap in care, we developed an mHealth app screening tool, NeuroScreen, to detect NCI that can be administered by all levels of clinical staff, including lay health workers.

**Objective:**

The purpose of this study was to examine sensitivity and specificity of an adapted version of NeuroScreen to detect NCI (as determined by a gold standard neuropsychological test battery administered by a trained research psychometrist) among HIV-infected South Africans when administered by a lay health worker.

**Methods:**

A total of 102 HIV-infected black South African adults who had initiated ART at least 12 months prior were administered NeuroScreen and a gold standard neuropsychological test battery in the participants’ choice of language (ie, English or isiXhosa). Three composite z scores were calculated for NeuroScreen: (1) sum of all individual test scores, (2) sum of all individual test scores and error scores from four tests, and (3) sum of four tests (abbreviated version). Global deficit scores were calculated for the gold standard battery where a score of 0.5 or greater indicated the presence of NCI.

**Results:**

The mean age of participants was 33.31 (SD 7.46) years, most (59.8%, 61/102) had at least 12 years of education, and 81.4% (83/102) of the sample was female. Gold standard test battery results indicated that 26.5% (27/102) of the sample had NCI. Sensitivity and specificity of age-, education-, and sex-adjusted NeuroScreen scores were 81.48% and 74.67% for composite score 1, 81.48% and 81.33% for composite score 2, and 92.59% and 70.67% for composite score 3, respectively.

**Conclusions:**

NeuroScreen, a highly automated, easy-to-use, tablet-based screening test to detect NCI among English- and isiXhosa-speaking South African HIV patients demonstrated robust sensitivity and specificity to detect NCI when administered by lay health workers. NeuroScreen could help make screening for NCI more feasible. However, additional research is needed with larger samples and normative test performance data are needed.

## Introduction

Neurocognitive impairment (NCI) is one of the most common sequelae and comorbid conditions of HIV infection and has significant medical, functional, and public health consequences. Prevalence estimates of NCI among South Africa’s 7 million people living with HIV (PLHIV) [1] range from 23% to 76% depending on whether the sample is antiretroviral therapy (ART) experienced or naïve, and do not differ between men and women [2,3]. These rates are similar to estimates in high-income countries [4-8]. Neurocognitive impairment in HIV, known as HIV-associated neurocognitive disorder (HAND) when HIV is determined to be the etiology, typically causes impairments in mental processing speed, learning, memory, attention and concentration, executive functions, and motor speed [6,9,10]. It ranges in severity from asymptomatic and mild forms to a severe dementia-type form [11]. Having even asymptomatic or mild NCI has been associated with increased risk for developing more severe impairment and mortality [12-16]. Neurocognitive impairment has also been associated with worse ART adherence (jeopardizing positive health outcomes), employment difficulties, impaired instrumental activities of daily living (eg, planning, driving, finance management), worse overall quality of life, and the need for more social services [12-23]. In addition, NCIs are also associated with poor decision making and greater HIV transmission risk behaviors (eg, unprotected sex) [17-19].

Screening for NCI in HIV is essential to good comprehensive care and treatment strategies [8,20,21]. As a first step in the clinical decision-making process, screening can enable providers to determine who is most likely to have NCI, detect early signs of NCI, allocate limited resources more effectively (eg, comprehensive neuropsychological testing, neurologic services, and social worker intervention), determine when to initiate and adjust ART regimens, track and monitor neurocognitive function, and educate patients about the impact of NCI and ways to minimize it—all which can improve health outcomes [22,23]. The impact of NCI on ART adherence can be minimized through comprehensive behavioral planning that incorporates reminder strategies and social support. Detecting NCI in highly infectious PLHIV (ie, those with detectable viral load) may help in tailoring transmission prevention strategies. Furthermore, if and when adjuvant pharmacotherapies or behavioral interventions become available for NCI in HIV, screening for NCI will be the first step to linking patients with these treatments. However, screening for NCI among PLHIV is not in widespread practice, and faces unique challenges in low- and middle-income countries and resource-limited settings. In South Africa, there are few locally developed screening tests for NCI in the local languages of those most affected by HIV, and a lack of expert personnel to administer them [13,24-28], missing critical opportunities to detect NCI and intervene for the millions of PLHIV there.

A variety of neurocognitive screening tests have been developed and evaluated to detect HAND, including computer-based and paper-and-pencil tests. Recent reviews of the most commonly used screening tests for HAND, mostly from high-income countries, indicated a wide range of sensitivity (<55%-90%) and specificity (70%-90%) depending on the test, the specific population, and type of NCI detected (ie, mild or HIV-associated dementia) [29]. Studies examining NCI screening tests used specifically with South African PLHIV found similarly wide variation in sensitivity and specificity depending on the test or combination of tests used [30,31]. None of these studies evaluated the performance of the screening tests when administered by nonspecialists or highly trained personnel. A screening test that can be administered by all levels of clinical staff is essential for scale-up in low- to middle-income countries and other resource-limited settings.

In South Africa, like many other low- and middle-income countries and resource-limited settings, lay health workers are utilized in clinical settings to provide essential HIV-related services (eg, AIDS education, HIV testing and counseling, ART adherence counseling, and preliminary management of mild mental disorders) that may not be otherwise available due to provider shortages [28,32-34]. Through task shifting, lay health workers work alongside nurses, physicians, pharmacists, and social workers. Lay health workers are ideally situated to provide a number of services to PLHIV, but certain routine tasks are still too complex, such as neurocognitive testing and screening [27].

To address this gap in HIV care, we developed NeuroScreen [35], an mHealth app for tablets designed to be used by all levels of health care personnel, including lay health workers, to screen for NCI. This novel software app for the Android operating system uses the touchscreen capabilities of tablet devices to highly automate neuropsychological testing. The neurocognitive screening test battery is embedded in a graphical user interface that automates test administration and allows for easy data management and reporting. All tests in NeuroScreen are automatically timed and scored—no hand scoring, score converting, or simultaneous and synchronized use of stopwatches is required. Tests that would normally require a pen or pencil use the touchscreen instead. To ensure that each administration is consistent, administrators are forced to sequence through all the standardized instructions. Furthermore, eight of the ten tests have audio-visual instructions, which are useful for low-literacy populations. Because NeuroScreen runs on a tablet and does not require an Internet connection, it is ultraportable and allows screenings to be administered in almost any location, such as remote or rural clinics or fast-paced and busy urban clinics requiring flexible use of examination rooms. Although computerized neuropsychological testing is becoming more common (but not so in resource-limited settings), mobile operating systems and devices are only just starting to be more commonly used as test delivery platforms.

In our previous work [35], we pilot tested a large-format mobile phone version of NeuroScreen in a small sample (N=44) of older (mean age 53.4 years) HIV-positive adults in the United States, most of whom (75%) had NCI as defined by a gold standard neuropsychological test battery. We also assessed acceptability of NeuroScreen among participants and 10 HIV providers. Evidence for construct validity of individual NeuroScreen tests as compared to the gold standard neuropsychological test battery was established. NeuroScreen also showed high sensitivity and moderate specificity to detect NCI at 93.94% and 63.64%, respectively. Both participants and providers indicated high acceptability for NeuroScreen. Providers remarked that NeuroScreen has potential to detect other disease-related NCIs.

The purpose of this study was to evaluate the ability of the lay health worker-administered NeuroScreen to detect NCI, as defined by a gold standard neuropsychological test battery. We examined the sensitivity and specificity, as well as the negative predictive value (NPV) and positive predictive value (PPV) of a South African-adapted NeuroScreen (for use with tablets and with the isiXhosa language) to detect NCI.

## Methods

This cross-sectional study adapted NeuroScreen from a large mobile phone screen format to 7-inch tablet format. Specifically, the Google Nexus 7 tablet was used for this study. Both the user interface and tests were updated to take advantage of the larger screen. NeuroScreen was also adapted for isiXhosa-speaking South Africans (the predominant Bantu language spoken in the Western Cape region of South Africa, where this study was conducted). Language translations underwent forward and backward translation, as well as vetting by the bilingual (English and isiXhosa) study staff. IsiXhosa translations were optimized to follow colloquial language conventions because it is the more predominantly used and understood form of isiXhosa in the communities where the study took place. Audio files were recorded with a native isiXhosa speaker for the audio-visual instructions. After all updates were made, the software engineers conducted quality assurance testing.

### Sample and Recruitment

A total of 102 HIV-positive black South African adults, aged 18 to 56 years, were recruited following their participation in a larger randomized controlled trial (RCT) of a multimedia, laptop-based, lay health worker-delivered ART-readiness intervention for ART initiators (known as Masivukeni or “Let’s Wake Up”) conducted in Cape Town, South Africa (see [36-38]). Inclusion criteria were (1) HIV-positive, (2) age 18 years or older, (3) willing to be contacted by study staff for participation in other research studies, (4) isiXhosa or English speaking, (5) capable of consent, (6) willing to complete 2 to 3 hours of neuropsychological testing, and (7) willing to allow access to medical records by study staff. Exclusion criteria were (1) not meeting one of the preceding criteria, and (2) presence of a current psychotic disorder, significant current suicidal ideation, and severe cognitive impairment precluding ability to give informed consent or participate based on clinical judgment of providers. All participants enrolled in the larger RCT were eligible to participate in this study. Ethics approval was obtained by the Human Research Ethics Committee at the University of Cape Town and the New York State Psychiatric Institute Institutional Review Board.

### Procedure

When the RCT participants completed their final study visit, the study nurse informed them about an additional study and requirements for participation. If a participant was interested and eligible, written informed consent was obtained. Then a trained lay health worker (two at each of the study clinics) administered NeuroScreen. A trained neuropsychology technician (one at each of the two study clinics) then administered the gold standard neuropsychological battery (approximately 3 hours). For participants who were unable to complete all study requirements on that visit, the neuropsychology technician scheduled another visit within 7 days to complete the gold standard neuropsychological battery. Neuropsychology technicians were blind to NeuroScreen results. Participants received the equivalent of US $40 for completion of all study procedures.

### Measures

#### Demographic and Neuromedical Data

Demographic and medical history data were available from the larger RCT. The psychometrist conducted an additional neuromedical questionnaire prior to administering the neuropsychological battery.

#### NeuroScreen

NeuroScreen [35] is comprised of 10 brief neuropsychological tests to briefly assess individuals across six neuropsychological domains most affected by HIV: verbal learning (two trials, five words) and memory (5-minute delayed recall); processing speed via two trail making test sequencing paradigms, two visual discrimination tasks, and a number input task; attention/concentration via a number span forward and backward task; executive functioning via an alternating trail making test sequencing paradigm; and motor functioning via a finger-tapping task (see Multimedia Appendix 1 for a complete description of each test). The tests are embedded in a graphical user interface that allows the administrator to enter patient data, administer tests, generate instant raw results, and save raw results to a secure and password-protected website and/or an internal storage card. After entering patient data, the administrator is required to read standardized test instructions or play videos that provide audio-visual instructions, is prompted at appropriate points to offer practice trials on selected tests, and is prompted to move on to the next test, thus sequencing through all the tests. NeuroScreen can be administered in English or isiXhosa. Once all data were transferred to the principal investigator’s secure and encrypted hard drive, all data were wiped from the device.

Total raw scores (eg, completion times, total correct) and error scores (eg, sequencing errors in the trail making tests and incorrect number inputs in the number input test) are systematically captured by the app. For this study, all raw scores were converted to z scores using the entire sample and timed tests were reverse scored so that higher scores indicated better performance. Three composite z scores were calculated: (1) sum of all individual test scores, (2) sum of all individual test scores and total errors from the trail making and number speed tests, and (3) sum of four tests (visual discrimination 1 and 2, trail making 1, and number span total).

#### Neuropsychological Evaluation

This paper-and-pencil neuropsychological battery assessed individuals across all neurocognitive domains using tests that are particularly sensitive to HIV-related NCI: learning and memory (Hopkins Verbal Learning Test-Revised [HVLT-R] [39] and the Brief Visuospatial Memory Test-Revised [BVMT-R] [40]); executive functioning (Color Trails Test 2 [41] and the Wisconsin Card Sorting Test [WCST] [42]); attention/concentration (Wechsler Memory Scales, Third Edition [WMS-III] Spatial Span [43] and Wechsler Adult Intelligence Scales, Third Edition [WAIS-III] Digit Span [44]); processing speed (Trail Making Test, Part A [45], Color Trails Test 1 [41], and WAIS-III Digit Symbol Coding and Symbol Search [44]); language (semantic fluency of animals, fruits, and vegetables [46]), and motor functioning (successive finger tapping and grooved pegboard [47]). This particular paper-and-pencil battery has been used in many HIV studies in the United States [7,26,48] and has been adapted for use with isiXhosa speakers in South Africa [2,3]. Data produced by this battery include (1) raw test scores, (2) T scores, (3) individual test deficit scores, and (5) global deficit scores (GDS).

A GDS was calculated for each participant and used to identify those with and without NCI. The GDS summarizes neuropsychological battery results by converting individual test T scores to a deficit score from 0 (no impairment) to 5 (severe impairment) [49]. Deficit scores are averaged across all tests to create the GDS. The GDS considers number and severity of impairments, assigning less weight to unimpaired performance and overcoming the disadvantage of averaging absolute performance, which gives equal weight to unimpaired and impaired scores [50]. The GDS method detects mild NCI (GDS≥0.5) across varying impairment patterns in different neurocognitive domains [49,51]. We set the threshold of GDS≥0.5 as indicative of NCI.

### Statistical Analysis

Univariate analyses were conducted to examine participant characteristics. To evaluate the ability of the lay health worker-administered NeuroScreen to detect NCI and evaluate its sensitivity and specificity, a logistic regression and receiver operator characteristic curve were used. First, the logistic regression model was used to establish a prediction model for NCI using the NeuroScreen score and age, gender, and education in the form of: log[*P(X)/* {1- *P(X)* }]= *β*_0_*+β*_1_*X*_1_*+β*_2_*X*_2_*+β*_3_*X*_3_*+β*_4_*X*_4_, where *P(X* *)* is the probability of having NCI given *X*=(*X*_1_,..., *X*_4_)=(patient’s age, sex, years of education, and NeuroScreen total score). After obtaining the estimated regression coefficients, the predicted probability of having NCI was calculated. After calculating the predicted probability for each patient, a receiver operating characteristic (ROC) analysis using the predicted probability and the gold standard NCI determination was calculated to evaluate the area under the curve (AUC) and to generate an optimal age-, education-, and sex-adjusted NeuroScreen score cutoff point to distinguish participants with NCI. The cutoff point was maximized by using the Youden index, which is equal to the sensitivity+specificity–1. Both PPV and NPV were computed for the optimized NeuroScreen cutoff score. All analyses were conducted using IBM SPSS version 23 (IBM Corp, Armonk, NY, USA).

## Results

### Sample Characteristics

As Table 1 shows, the sample was predominantly female with a mean age of 33.31 (SD 7.46) years and most (59.8%, 61/102) had at least 12 years of education. Although 30 participants reported being held back for at least one grade during their schooling, only nine participants reported receiving special classes to assist them with learning difficulties (the South African education system does not routinely assess for and diagnose learning disorders). Four participants reported having had a loss of consciousness greater than 15 minutes (for head injury and other medical issues). One participant reported having a stroke that resulted in numbness in the right arm, three participants reported having had one seizure, and one participant reported being diagnosed with epilepsy at age 10. Four participants reported taking medication for high blood pressure.

### NeuroScreen Performance

Table 2 displays raw mean scores for all NeuroScreen tests, as well as the three composite total *z* scores. On average, participants were able to learn 8.45 (SD 1.51) words across two learning trials (same five words per trial) and recall 3.39 (SD 1.20) words after a 5-minute delay. The mean total number span backward and forward score was 5.87 (SD 1.29) with a maximum possible score of 17. The mean total correct responses on Visual Discrimination 1 was 11.28 (maximum=61) and 25.97 on Visual Discrimination 2 (maximum=150). The mean completion time on Number Input Speed was 45.02 (SD 18.08) seconds. Mean completion times for Trails Test 1 was 18.93 (SD 18.17) seconds, 31.95 (SD 21.21) seconds for Trails Test 2, and 18.81 (SD 16.32) seconds for Trails Test 3. The mean total finger taps for both the dominant and nondominant hand was 454.73 (SD 60.03) taps across five trials.

### Gold Standard HIV Neuropsychological Battery Performance

Results from the full neuropsychological battery (Table 3) indicated that the sample had a mean global T score of 48.01 (SD 4.79). Table 3 also displays performance (adjusted T scores) across the individual tests. The mean GDS was 0.36 (SD 0.40) and 26.5% (27/102) had NCI using a GDS score of 0.5 or greater to indicate impairment.

### Sensitivity and Specificity

#### NeuroScreen Total Score 1 (Sum of All Tests)

**Figure 1:**
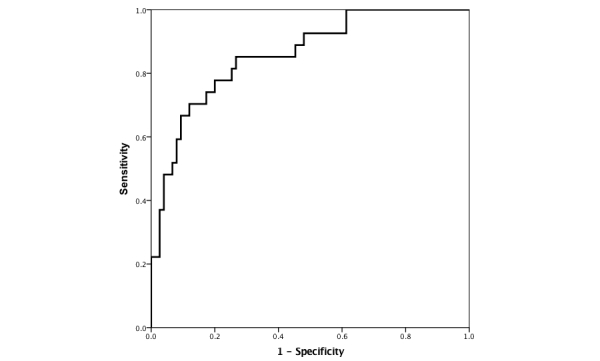
Receiver operating characteristic curve for NeuroScreen total score 1.

Using the logistic model with the first NeuroScreen total score adjusted for age, education, and sex to predict the gold standard NCI in the ROC analysis, the AUC was 0.86 (95% CI 0.78-0.94; see Figure 1). The Youden index NeuroScreen predicted NCI cut-score of 0.21 maximized sensitivity at 81.48% (95% CI 61.92%-93.70%) and specificity at 74.67% (95% CI 63.30%-84.01%). The PPV was 53.66% and the NPV was 91.80%. Using this cut-score yielded 19 false positives and 5 false negatives. The mean completion time for all the tests was 23.88 (SD 6.21) minutes.

#### NeuroScreen Total Score 2 (Sum of All Tests and Available Error Scores)

**Figure 2:**
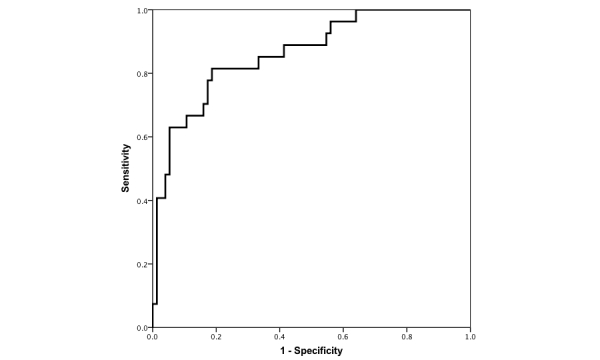
Receiver operating characteristic curve for NeuroScreen total score 2.

An ROC analysis using the logistic model with the second NeuroScreen total score to predict NCI had an AUC of 0.86 (95% CI 0.78-0.94; see Figure 2). The Youden index maximal sensitivity was 81.48% (95% CI 61.92%-93.70%) and specificity was 81.33% (95% CI 70.67%-89.40%). The PPV was 61.11% and the NPV was 92.42%. Using this cut-score yielded 14 false positives and 5 false negatives. The mean completion time was the same as the preceding.

**Table 1:** Sample characteristics (N=102).

Characteristic	Participants	Min	Max
Age (years), mean (SD^a^)	33.31 (7.46)	19	56
Gender (female), n (%)	83 (81)	—	—
Education (years completed), mean (SD)	11.25 (1.99)	3	14
Traumatic brain injury with loss of consciousness >15 minutes, n (%)	4 (4)	—	—
Likely learning difficulty, n (%)	9 (9)	—	—
Most recent CD4 cell count, mean (SD)^b^	501.31 (287.41)	47	1654
Most recent viral load undetectable, n (%)^c^	81 (91)	—	—

^a^SD: standard deviation.

^b^CD4: cluster of differentiation 4. CD4 cell count available for 88 participants.

^c^Viral load data available for 81 participants.

**Table 2:** NeuroScreen performance (raw).

Test	Mean (SD^a^)	Min	Max
Finger tapping total (both hands)	454.73 (60.03)	190.00	581.00
Visual discrimination 1 total correct	11.28 (3.67)	4.00	19.00
Visual discrimination 2 total correct	25.97 (7.97)	1.00	48.00
Number span total (forward and backward)	5.87 (1.29)	3.50	9.50
Verbal learning total correct	8.45 (1.51)	3.00	10.00
Delayed verbal recall total correct	3.39 (1.20)	0.00	5.00
Trail making 1 completion time (seconds)^b^	–18.93 (18.17)	–120.00	–6.06
Trail making 2 completion time (seconds)^b^	–31.95 (21.21)	–120.00	–11.17
Trail making 3 completion time (seconds)^b^	–18.81 (16.32)	–120.00	–5.05
Number speed completion time (seconds)^b^	–45.02 (18.08)	–117.75	–23.60
Full battery completion time (minutes)	23.88 (6.21)	9.00	52.00

^a^SD: standard deviation.

^b^Indicates reverse scored (slower time=worse performance).

**Table 3:** Gold standard neuropsychological test battery performance (adjusted).

Test		Mean (SD^a^)	Min	Max
Global T		48.01 (4.79)	34.00	57.63
**Motor functioning tests**				
	Successive finger tapping (dominant hand)	46.51 (11.29)	–1.31	64.05
	Successive finger tapping (nondominant hand)	44.98 (13.45)	–43.87	61.66
	Grooved pegboard (dominant hand)	47.41 (7.96)	12.67	60.05
	Grooved pegboard (nondominant hand)	49.26 (3.09)	34.09	53.27
Hopkins Verbal Learning Test-Revised				
	Total trials 1-3	45.97 (8.33)	24.34	62.67
	Delay recall total	44.74 (9.86)	22.64	67.13
**Brief Visuospatial Memory Test-Revised**				
	Total Trials 1-3	48.40 (9.80)	28.09	73.75
	Delay Total Recall	49.58 (11.48)	29.03	72.20
**Wechsler Adult Intelligence Scales, Third Edition (WAIS-III)**				
	Digit symbol coding total	46.73 (10.08)	25.45	77.15
	Symbol search total	47.24 (8.95)	27.19	64.76
	Wechsler Memory Scales, Third Edition spatial span total	50.47 (9.65)	30.06	73.85
**Processing speed tests**				
	Trail making test, part A	43.82 (11.99)	–7.48	72.04
	Color trails test 1	46.72 (9.98)	12.22	64.02
	Color trails test 2	48.32 (9.73)	14.83	66.80
	WAIS-III Digit span total	49.33 (1.21)	46.70	52.09
**Wisconsin Card Sorting Test**				
	Perseverative errors	50.74 (12.89)	3.52	63.76
	Trials to first sort	48.10 (11.32)	29.66	57.98
	Failures to maintain set	50.59 (4.79)	31.88	53.94
**Language tests**				
	Animal fluency total	49.41 (8.70)	27.72	70.48
	Fruit and vegetable fluency total	51.83 (8.75)	33.26	72.21

^a^SD: standard deviation.

**Figure 3:**
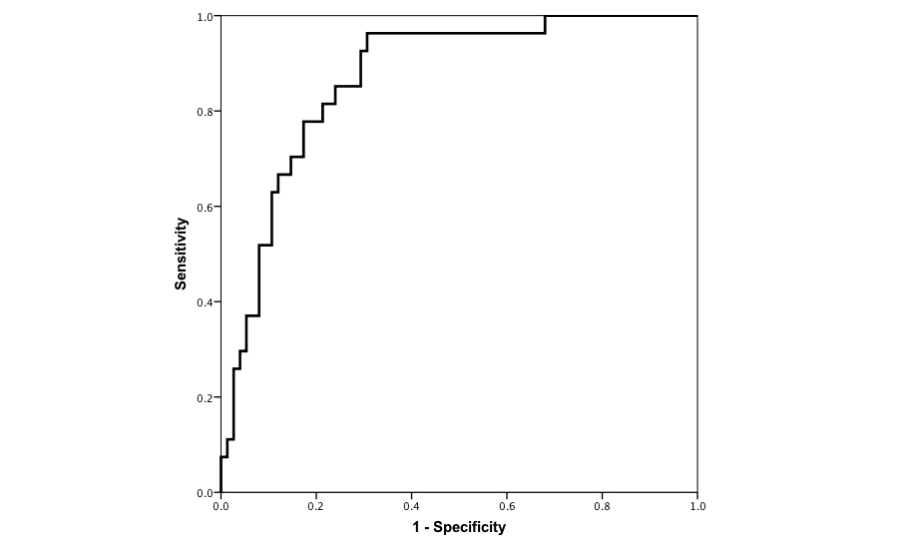
Receiver operating characteristic curve for NeuroScreen total score 3 (abbreviated version).

#### NeuroScreen Total Score 3 (Sum of Four Tests)

Using the logistic model with the third NeuroScreen total score with age, education, and sex to predict the gold standard NCI in the ROC analysis, the AUC was 0.87 (95% CI 0.80-0.94; see Figure 3). The Youden index NeuroScreen predicted NCI cut-score of 0.18 maximized sensitivity at 92.59% (95% CI 75.71%-99.09%) and specificity at 70.67% (95% CI 59.02%-80.62%). The PPV was 53.19% and the NPV was 96.36%. Using this cut-score yielded 22 false positives and 2 false negatives. The estimated completion time of these four tests was approximately 12 minutes.

## Discussion

In a sample of 102 HIV-positive predominantly female South Africans on ART for at least 1 year, gold standard neuropsychological test results indicated 26% of the sample had at least mild NCI. This rate of NCI is consistent with other research from South Africa [3], but lower than NCI rates found in the United States [48] among ART-experienced PLHIV. NeuroScreen—when administered by a lay health worker—had robust test characteristics to detect gold standard-defined NCI ranging from 81% to 93% sensitivity and 71% to 81% specificity, depending on the combination of NeuroScreen test scores used. Our study provides preliminary evidence for the validity of NeuroScreen to detect NCI among English- and isXhosa-speaking South African adults living with HIV. NeuroScreen shows promise as an easy-to-use, brief NCI screening test that can be administered by lay health workers, and likely most levels of health care staff.

Deciding which NeuroScreen total score to use must be weighed by each provider and/or health care system. Given estimated rates of NCI that exist among PLHIV in South Africa (23%-76% [2,3]), the extra burden screening for NCI places on an already overburdened and resource-limited health care system must be weighed against the consequences misdetection might have on patients and the health care system. Ideally, anyone who screens positive for NCI would be referred for confirmatory testing with a comprehensive neuropsychological assessment. However, doing so is simply not feasible in many settings, such as South Africa. For resource-limited settings, such as South Africa, it may make sense to use the shortest version of NeuroScreen (12 minutes to administer with 92.59% sensitivity and 70.67% specificity) even though the rate of false positives would be higher than using the full NeuroScreen with error scores (25-minute administration time with 81.48% sensitivity and 81.33% specificity). The consequences of a positive screen (eg, extra treatment planning considerations and additional ART adherence support) might outweigh the consequences of missing someone who truly has NCI (eg, poor adherence/health outcomes).

Although the full version with error scores is approximately twice as long to administer than the abbreviated version, its specificity is much higher. As a potential substitute for a full neuropsychological assessment (which is not feasible and highly unlikely to occur in this setting), 25 minutes of a lay health worker’s time is much less resource intensive than 3 to 5 hours of a neuropsychologist’s, psychologist’s, and/or neuropsychology technician’s time to administer a neuropsychological test battery and score it. Although we do not believe a short battery of tests such as NeuroScreen should replace gold standard neuropsychological assessments, it may help provide clinics in resource-limited settings that do not have access to the gold standard measures and procedures with a viable alternative. Having a screening test that can be administered by any staff, lay health worker included, can help clinics identify those PLHIV at highest risk for having NCI and make important treatment recommendations and referrals to address it.

Compared to other computerized and paper-and-pencil screening tests for NCI in HIV, NeuroScreen performed similarly to and better than some tests (see [29]). Compared to screening tests specifically evaluated in South Africa [30,31], NeuroScreen also yielded robust sensitivity and specificity across all three total scores. Moreover, these robust performance characteristics were achieved by NeuroScreen when administered by a lay health worker. NeuroScreen, to our knowledge, is the only computerized, mHealth neurocognitive screening test tablet app for NCI detection in South Africa for English and isiXhosa speakers. Furthermore, it is the only such test designed to be administered by all levels of clinical staff with minimal training and supervision.

It is important to note that there was discrepancy between NeuroScreen and the gold standard neuropsychological battery in defining who had NCI. Overall, NeuroScreen indicated that more participants had NCI than did the gold standard battery. The discrepancy could be explained in part by NeuroScreen’s tests being more difficult than the gold standard tests (ie, floor effects on NeuroScreen tests) or practice effects. We did not randomize test sequence administration—all participants were administered NeuroScreen first then the gold standard tests. Some participants’ performances on the gold standard tests could have benefited from first taking the NeuroScreen tests. If the benefit was big enough, performance on the gold standard tests could have appeared within the normal range (ie, not impaired). Additionally, the discrepancy could also be due to human factors involved in the gold standard battery administration, such as time keeping and recording errors, or subtle biases in test scoring (although our neuropsychology technicians received intensive training and ongoing supervision). There could also be issues regarding language—more participants opted to take NeuroScreen in English (n=27) than the gold standard battery (n=6). Further research is needed to fully understand this discrepancy.

It is important to note this study’s limitations. First, we had a small sample of PLHIV in South Africa to evaluate NeuroScreen’s sensitivity and specificity that was mostly female. Second, we did not formally assess language fluency, for either English or isiXhosa. Participants were asked for their language preference by the lay health worker. Similarly, the psychometrist discussed with the participant in which language they would like to take the gold standard battery. Third, the NCI detected in this study may or may not be a result of HIV—numerous factors can cause and/or contribute to the development of NCIs, such as low education, head injuries, and other medical factors (many of which were observed in this sample). Fourth, normative performance data have not been established for NeuroScreen among isiXhosa-speaking South Africans, or other South African language groups, making generalization of performance on it inappropriate. Finally, the gold standard battery had numerous neuropsychological tests assessing neurocognitive domains that NeuroScreen did not assess (verbal fluency and perseveration), although the tests in NeuroScreen were specifically chosen to assess those neurocognitive domains most typically affected by HIV. Furthermore, NeuroScreen is not meant to be a substitute for a thorough neuropsychological assessment.

Despite these limitations, we believe NeuroScreen has potential to offer busy health clinics and research studies with a brief, easy-to-use solution to screen for NCI among PLHIV, and among patients with other brain-involving diseases and disorders. With a tool such as NeuroScreen, better referrals, tracking, and integration with electronic medical records could be achieved. However, more research is needed to validate NeuroScreen as a screening tool for NCI. A larger sample, statistically powered to establish internal and external validity indicators, is essential for NeuroScreen scale-up.

Computerized neurocognitive testing and screening are transforming clinical practice for neuropsychological assessments. Mobile technology offers a powerful platform that is ultraportable and can be easy to use. This study provides evidence that our app, NeuroScreen, has clinically useful psychometric properties to detect NCI when administered by lay health workers. Taking advantage of mobile platforms and automating many components of the neurocognitive testing process may help to make testing more accurate, efficient, affordable, and accessible to those who need testing, especially in resource-limited settings.

## Appendix

Multimedia Appendix 1 NeuroScreen test descriptions.

## Abbreviations

ART: antiretroviral therapy
AUC: area under the curve
BVMT-R: Brief Visuospatial Memory Test-Revised
GDS: global deficit score
HAND: HIV-associated neurocognitive disorder
HVLT-R: Hopkins Verbal Learning Test-Revised
NCI: neurocognitive impairment
NPV: negative predictive value
PLHIV: people living with HIV
PPV: positive predictive value
RCT: randomized controlled trial
ROC: receiver operator characteristic
WAIS-III: Wechsler Adult Intelligence Scales, Third Edition
WCST: Wisconsin Card Sorting Test
WMS-III: Wechsler Memory Scales, Third Edition
