# Supplementary material for: A Mobile App to Screen for Neurocognitive Impairment: Preliminary Validation of NeuroScreen Among HIV-Infected South African Adults
Source: JMIR Mhealth Uhealth. 2018 Jan 5;6(1):e5. doi: 10.2196/mhealth.9148 (PMC5775487; doi:10.2196/mhealth.9148)
Supplement: Multimedia Appendix 1 [file mhealth_v6i1e5_app1.pdf]

Verbal Learning and Delayed Recall (Memory)

Learning Paradigm

Structure: 5 word list (English and isiXhosa)
Trials: 2 immediate recall trials
Raw Data Captured: Total correct words recalled per trial
Total repetitions recalled per trial
Total number of intrusions
Sequence of words recalled
Time to complete task
Scores: Total correct words across both trials

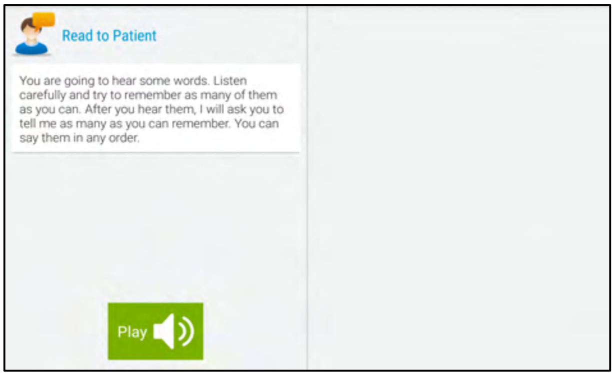

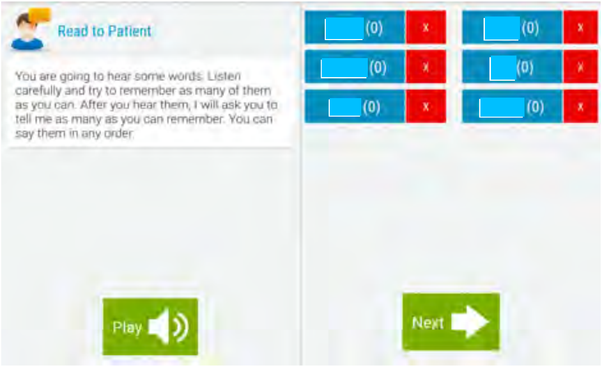

Test administrator reads test instructions to patient. When the "Play Audio" button is pressed, the patient hears the word list. All words are spoken in a highly enunciated, standardized voice at 2-second intervals.	After the word list is played, the word choices appear on the screen. If the "Play Audio" button is accidently pressed again, a pop-up warning appears asking if the word list should be played again, even though it was just played.	The test administrator records the words the patient says by tapping the appropriate button. Words can be counted more than once (repetitions); intrusions are captured using the 'OTHER' button.

Delayed Recall/Memory Paradigm (5-minute Delay)

Structure: ~5-minute delay (automatically cued during administration)
Trials: Free recall, cued recall for words missed on free recall trial, multiple choice recall for words missed on cued recall trial
Raw Data Captured: Total correct words recalled per each trial
Total repetitions recalled for free recall trial
Total number of intrusions recalled for free recall trial
Sequence of words recalled for free recall trial
Time to complete task
Actual delay time
Scores: Total correct words for free recall

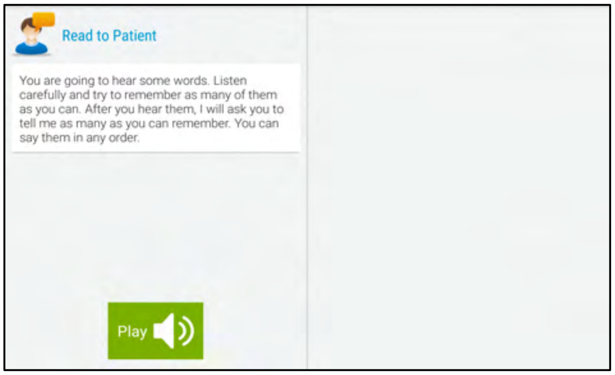

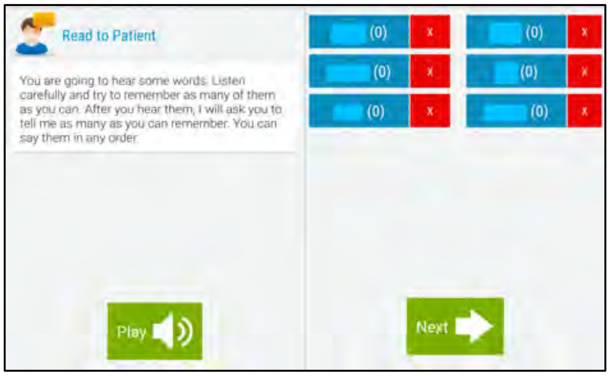

After an approximate 5-minute delay (the test is cued automatically after 5-minutes, but will let the administrator complete whatever task currently on), the test administrator reads test instructions to patient.		The test administrator records the words the patient says by tapping the appropriate button. Words can be counted more than once (repetitions); intrusions are captured using the 'OTHER' button.

Working Memory

Structure: Immediate recall of increasing spans of numbers (min. span=3 numbers; max. span=8 numbers)
Trials: Forward spans and backward spans; two attempts per span; trial ends with two incorrect recalls on one span
Raw Data Captured: Numbers recalled per span
Number of attempts per span
Total correct spans per trial
Time to complete each span
Scores: Total correct spans forwards
Total correct spans backwards

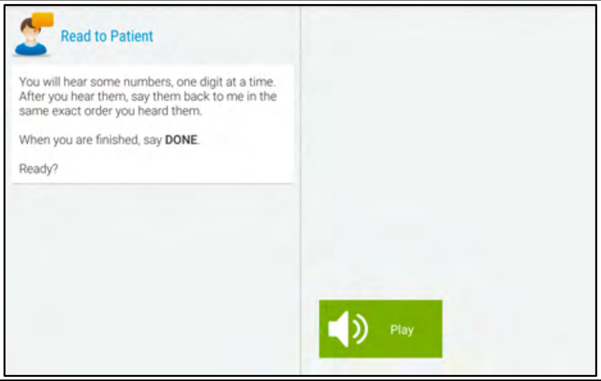

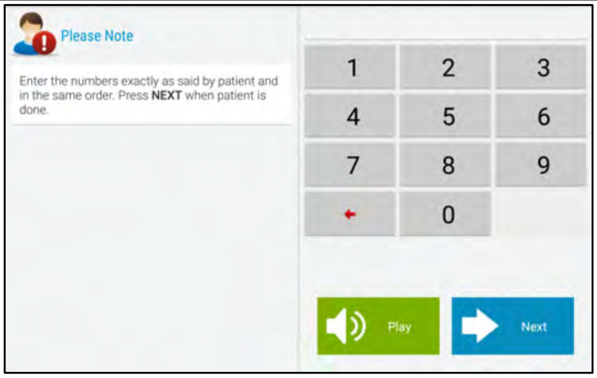

The test administrator reads the instructions to the patient, and then when ready, presses the “Play” button. The number sequences will be played via audio file at exactly 1-second per number.	Only after the number sequence has played can the test administrator move to the next screen.	The test administrator enters the numbers the patient says in the same order on the keypad. When the patient is done, the test administrator presses the green phone button. If the patient’s response was correct, then the next number sequence is presented. If the patient’s response was incorrect, they hear the same sequence again.<br><br>If the test administrator incorrectly presses a wrong button during patient sequence recall, the red back arrow button can be used to delete the last entry.

Executive Functioning

Trail Making Paradigm

Structure: Trail making paradigm-alternating numbers and colors
Trials: Practice and actual
Raw Data Captured: Latency from test start to first interaction with tablet
Number of times finger lifted off screen
Total sequencing errors made
Time between each correct circle
Sequence of circles and errors
Scores: Completion time
Total sequencing errors made

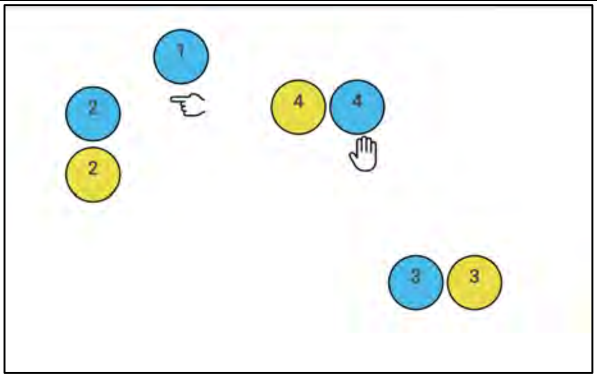		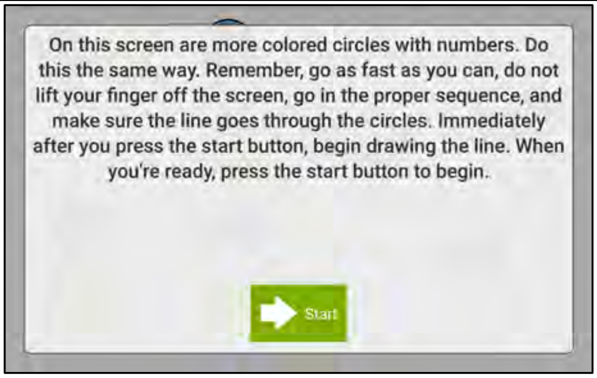

The patient is given the device and shown an audiovisual instruction video demonstrates the task. An animated hand visually instructs the patient what to do while audio instructions are played concurrently.	The patient is given a trial to practice connecting the circles in the right order.	The patient must use a finger to draw a line between the circles alternating between colors order. If the sequence is violated, the patient receives an error message and is instructed to go back to the last circle and continue in the correct order.

Number Speed Paradigm

Structure: Enter increasingly longer number sequences into the keypad as fast as possible.
Trials: Actual (no practice); Starting at sequence of 3 numbers up to sequence of 9 numbers
Raw Data Captured: Latency from test start to first interaction with tablet
Time to complete correct sequence
Total number input errors
Error specificity (wrong number inputted)
Input time for each number (quasi-reaction time)
Scores: Completion time per trial
Total completion of all trials

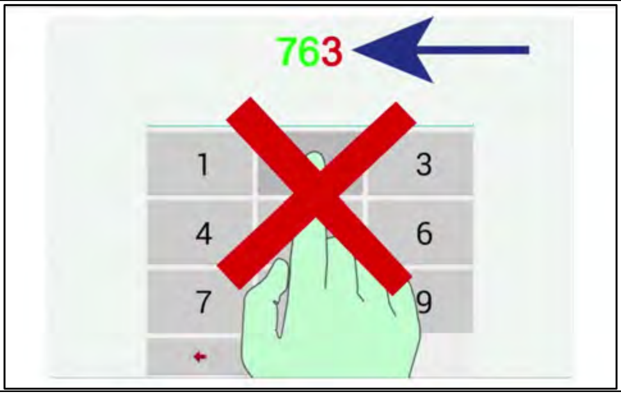		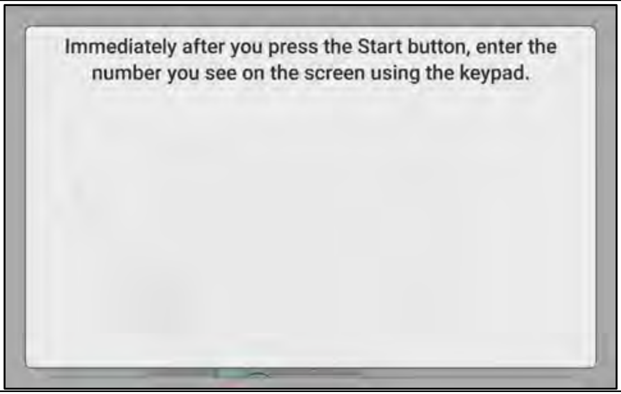

The patient is given the device and shown an audiovisual instruction video that demonstrates how to do the task. An animated hand visually instructs the patient what to do while audio instructions are played concurrently.		The patient must use a finger to enter the sequence into the keypad. If the patient enters an incorrect number, it will turn red. The patient must notice it and then correct it before the test moves on to the next number sequence.

Attention

Trail Making Paradigm

Structure: Trail making paradigm-numbers only

Trials: Practice and actual

Raw Data Captured: Latency from test start to first interaction with tablet
Number of times finger lifted off screen
Total sequencing errors made
Time between each correct circle
Sequence of circles and errors

Scores: Completion time
Total sequencing errors made

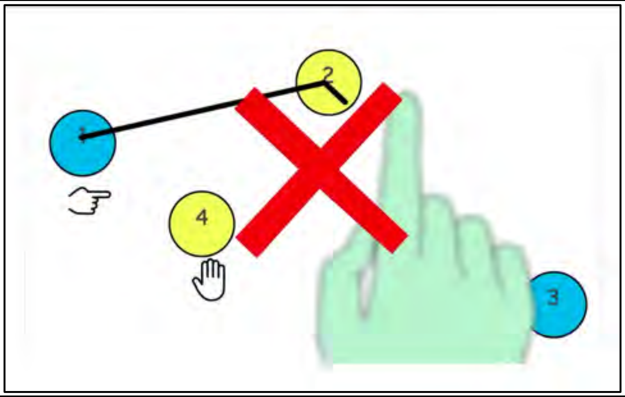

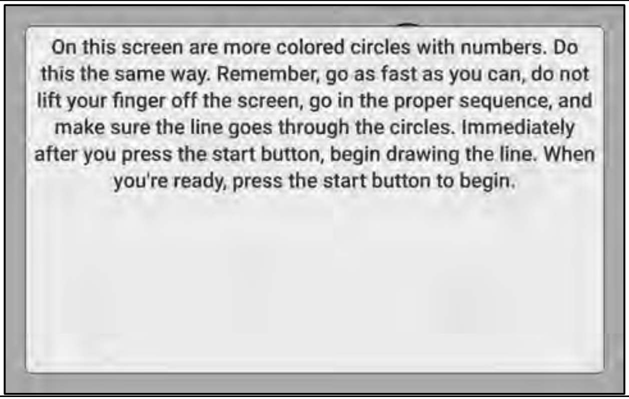

The patient is given the device and shown an audiovisual instruction video that demonstrates how to do the task. An animated hand visually instructs the patient what to do while audio instructions are played concurrently.	The patient is given a trial to practice connecting the circles in the right order.	The patient must use a finger to draw a line between the circles in the correct sequence. If the sequence is violated, the patient receives an error message and is instructed to go back to the last circle and continue in the correct order.

Processing Speed

Visual Discrimination 1

Structure: Symbol matching
Trials: Practice and 45-second actual
Raw Data Captured: Latency from test start to first interaction with tablet
Actual response for each item
Total number of correct responses
Total number of incorrect responses
Input time for each item (quasi-reaction time)
Scores: Total correct
Total incorrect

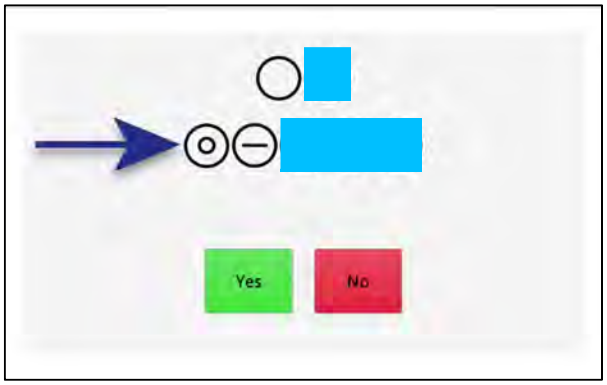

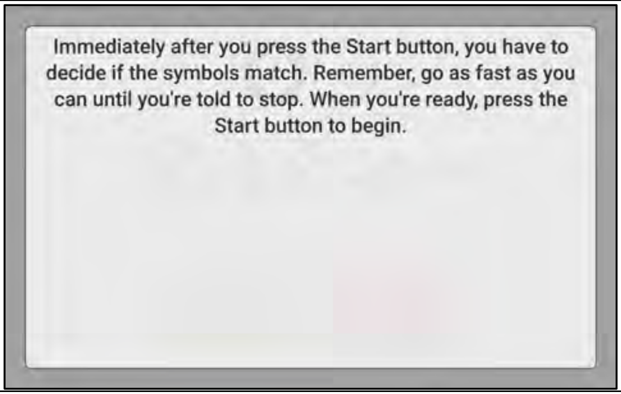

The patient is given the device and shown an audiovisual instruction video that demonstrates how to do the task. An animated hand visually instructs the patient what to do while audio instructions are played concurrently.	The patient is given a trial to practice with automated feedback.	The patient must use decide if one of the symbols any of the symbols in the bottom row and press the Yes or No button when decided.

Visual Discrimination 2

Structure: Symbol and number matching
Trials: Practice and 45-second actual
Raw Data Captured: Latency from test start to first interaction with tablet
Actual response for each item
Total number of correct responses
Total number of incorrect responses
Input time for each item (quasi-reaction time)
Scores: Total correct
Total incorrect

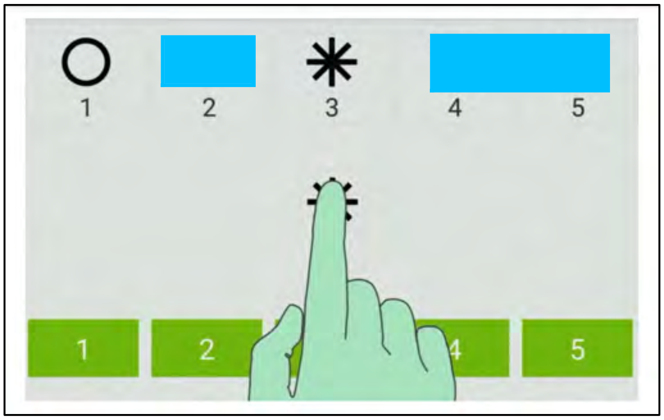

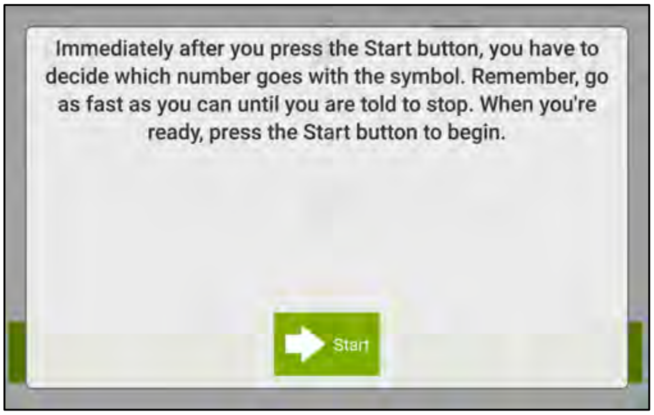

The patient is given the device and shown an audiovisual instruction video that demonstrates how to do the task. An animated hand visually instructs the patient what to do while audio instructions are played concurrently.	The patient is given a trial to practice with automated feedback.	The patient must use decide which number goes with the symbol in the middle of screen and press the corresponding number button at the bottom of the screen.

Motor Speed

Structure: Finger tapping
Trials: 5 10-second trials per hand starting with dominant hand (hands switch after 3 trials)
Raw Data Captured: Latency from test start to first interaction with tablet
Number of false start taps (impulsivity)
Total taps per trial
Total taps per second within each trial (performance curve)
Scores: Total taps per hand

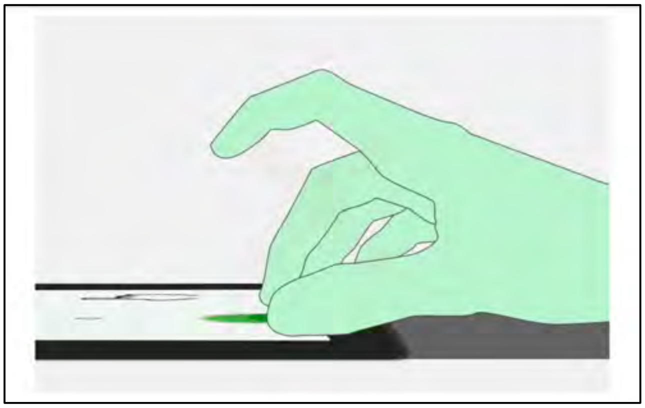

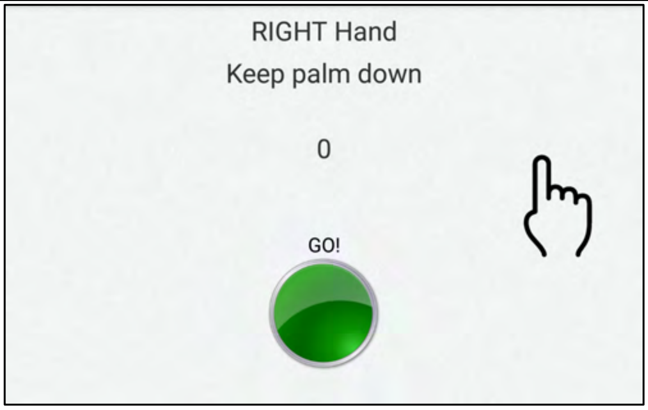

The patient is given the device and shown an audiovisual instruction video that demonstrates how to do the task. An animated hand visually instructs the patient what to do while audio instructions are played concurrently.		The patient taps the button as fast as possible when it turns green.
